# Facile synthesis of α-Fe_2_O_3_ nanodisk with superior photocatalytic performance and mechanism insight

**DOI:** 10.1088/1468-6996/16/1/014801

**Published:** 2015-01-16

**Authors:** Yang Huang, Dahu Ding, Minshen Zhu, Wenjun Meng, Yan Huang, Fengxia Geng, Jie Li, Jing Lin, Chengchun Tang, Zhongfang Lei, Zhenya Zhang, Chunyi Zhi

**Affiliations:** 1 Department of Physics and Materials Science, City University of Hong Kong, 83 Tat Chee Avenue, Kowloon, Hong Kong; 2 College of Resources and Environmental Sciences, Nanjing Agricultural University, Nanjing 210095, People’s Republic of China; 3 Graduate School of Life and Environmental Science, University of Tsukuba, Tsukuba, Ibaraki 305-8572, Japan; 4 College of Chemistry, Chemical Engineering and Materials Science, Soochow University, Suzhou 215123, People’s Republic of China; 5 School of Materials Science and Engineering, Hebei University of Technology, Tianjin 300130, People’s Republic of China; 6 Shenzhen Research Institute, City University of Hong Kong, Shenzhen, People’s Republic of China

**Keywords:** *α*-Fe_2_O_3_, microwave-assisted synthesis, photocatalytic activity, active species

## Abstract

Intrinsic short hole diffusion length is a well-known problem for *α*-Fe_2_O_3_ as a visible-light photocatalytic material. In this paper, a nanodisk morphology was designed to remarkably enhance separation of electron-hole pairs of *α*-Fe_2_O_3_. As expected, *α*-Fe_2_O_3_ nanodisks presented superior photocatalytic activity toward methylene blue degradation: more than 90% of the dye could be photodegraded within 30 min in comparison with a degradation efficiency of 50% for conventional Fe_2_O_3_ powder. The unique multilayer structure is thought to play a key role in the remarkably improved photocatalytic performance. Further experiments involving mechanism investigations revealed that instead of high surface area, ·OH plays a crucial role in methylene blue degradation and that O^·2−^ may also contribute effectively to the degradation process. This paper demonstrates a facile and energy-saving route to fabricating homogenous *α*-Fe_2_O_3_ nanodisks with superior photocatalytic activity that is suitable for the treatment of contaminated water and that meets the requirement of mass production.

## 1. Introduction

Environmental problems are caused mostly by the arbitrary disposal of waste and toxic pollutants without proper treatment. Increasingly severe water pollution is especially harmful to human health. To address this issue, a number of processes have been adopted to remove pollutants from wastewater, including adsorption, flocculation, oxidation, and reduction. Among these methods, photocatalysis, a green technology, shows great potential for the elimination of organic pollutants from wastewater. It has been reported that a number of semiconductors can degrade a variety of organic pollutants under the irradiation of light of different wavelengths [1–6]. For example, TiO_2_, with a bandgap of ∼3.2 eV, is well recognized as a perfect UV photocatalyst [7]. However, the lack of response to visible light limits its application. Consequently, reduction of the TiO_2_ bandgap has attracted much attention recently [8–13]. Nevertheless, considering the fact that visible light accounts for 46% of the solar energy, it is preferable to develop a semiconductor that shows response in the visible-light region. Thanks to the untiring efforts of researchers, various kinds of visible light-driven photocatalysts have been developed, and many of them exhibit good photodegradation ability with respect to different organic pollutants [14, 15]. But most of these photocatalysts need a complicated and strict synthesis process, which incurs a high synthesis cost. Some of them are not so stable under ambient conditions. All these negative aspects make them far from practical applications.

Hematite (*α*-Fe_2_O_3_), which is the most stable iron oxide under ambient conditions, can absorb visible light since it has a narrow bandgap (*E*_*g*_ = 2.1 eV). This makes it a potential competitive candidate for a visible light–driven photocatalyst [16]. However, because of its intrinsic short hole diffusion length (2–4 nm) [17, 18], the photogenerated electron-hole pairs in *α*-Fe_2_O_3_ can recombine easily. In view of enhancing the separation of the electron-hole pairs, a number of nanostructures, such as rods [19], cubes [20], sheets [21], plates [22], flowers [23], and rings [24], have been fabricated by various methods, including sol-gel, solvothermal, hydrothermal, and microwave-assisted methods. Among these methods, microwave-assisted chemistry is a promising technique which is based on the interactions between electric dipoles in dielectric materials, liquids, or solid materials and an applied electromagnetic field [25]. Due to shorter processing time, rapid heating, and the formation of homogeneous products, the reaction time and energy cost can be reduced significantly [26, 27]. Moreover, a microwave system that was recently designed can control exact on-line determination of the reaction temperature and pressure inside a reaction vessel, which is not achieved through traditional methods. All these attractive features lead to a growing interest in such a novel microwave-assisted processing method.

Thus a nanodisk morphology was designed to improve the generation and separation of electron-hole pairs in *α*-Fe_2_O_3_ for enhanced photocatalytic performance. Uniform *α*-Fe_2_O_3_ nanodisks were successfully prepared by a microwave-assisted hydrothermal method. By taking advantage of microwave assistance, the synthesis time was greatly reduced to 15 min. In addition, with the unique multilayer structure, superior photocatalytic degradation of methylene blue (MB) under visible light was obtained. Furthermore, the mechanism of photodegradation was investigated profoundly. Through the help of powerful scavengers, hydroxyl radicals (·OH) were found to be the main active species in the *α*-Fe_2_O_3_ nanodisk degradation system, and superoxide radical anions (O^·2−^) are also thought to contribute to the degradation process. This paper highlights the promising application of *α*-Fe_2_O_3_ nanodisks in pollutant elimination via a rapid and green microwave-assisted synthesis method.

## 2. Experimental details

### 2.1. Preparation of Fe_2_O_3_ nanodisk

All the chemicals were analytical grade and were used without further purification. The synthesis process is described as follows. Briefly, 121.7 mg of FeCl_3_ and 106.6 mg of Na_2_SiO_3_ · 9H_2_O were dissolved in 30 mL and 20 mL of deionized water (DW), respectively. Then the solutions were mixed and sonicated for 10 min to form a transparent orange solution. After that, the homogeneous mixture was transferred into a 100 mL Teflon-lined double-walled digestion vessel and treated at a controllable temperature (140 °C, 160 °C, 180 °C, and 200 °C) for 30 min in a microwave digestion system (MDS-6G, Shanghai Sineo Microwave Chemistry Technology Co., Ltd). After the vessel was taken out and cooled to room temperature, the product was collected by centrifugation and rinsed with DW and absolute ethanol several times. Finally, samples were dried overnight in an oven at 80 °C [28]. In addition, the effects of microwave irradiation time and additional annealing treatment were investigated in this study, as listed in table S1 (Supporting Information).

### 2.2. Characterization

Powder x-ray diffraction (XRD) patterns were recorded on a BRUKER D2 PHASER diffractometer, which was equipped with CuK_*α*_ irradiation (*λ* = 1.541 84 Å) and operated at 10 mA and 30 kV. X-ray photoelectron spectroscopy (XPS) data were obtained with an ESCALAB 220i-XL electron spectrometer from VG Scientific using AlK_*α*_ irradiation. The morphology was investigated by environmental scanning electron microscope (ESEM, FEI/Philips XL30), transmission electron microscope (TEM, FEI/Philips Tecnai 12 Bio TWIN), and high-resolution transmission electron microscope (HR-TEM, Jeol 2010). UV/vis spectra of aqueous samples were recorded on a Dynamica HALO DB 20 spectrophotometer. UV/vis diffuse reflectance spectra (DRS) of solid samples were recorded on a Shimadzu UV-3600 UV–vis-NIR spectrophotometer equipped with an integrating sphere attachment. BaSO_4_ was used as the background, and the range was 300–800 nm as determined by the DRS scan. Fluorescence spectra were recorded on a VARIAN CARY Eclipse fluorescence spectrophotometer. N_2_ adsorption/desorption isotherms at 77 K were measured by using an adsorption instrument (TriStar 3000) to evaluate the pore structures and surface area. Electrochemical impedance spectroscopy (EIS) measurements were performed on a CHI 760e electrochemical workstation (CH Instruments Company) with a conventional three-electrode system (Fe_2_O_3_ as the working electrode, Pt as the counter electrode, and Ag/AgCl as the reference electrode) in 1 M KOH. The details of the EIS measurements are included in the supporting information (available at stacks.iop.org/STAM/16/014801/mmedia).

### 2.3. Photocatalysis experiments

MB, which is a common organic pollutant resulting from industrial production of dyes, is adopted in this study to evaluate the photocatalytic activities of commercial (Alfa Aesar, analytical grade, as a reference) and as-synthesized *α*-Fe_2_O_3_ samples. Visible light-driven photodegradation tests were conducted in a 100 mL customized vessel surrounded by a circulating water jacket and maintained at 23–26 °C under ambient conditions. A 300 W Xe lamp equipped with a 420 nm low-pass filter was used as the illuminating source. This was positioned 10 cm away from the liquid level of the MB aqueous solution, providing a light intensity of ∼3.77 mW cm^−2^. During the photodegradation experiments, the sample (10 mg) was dispersed in MB aqueous solution (50 mL) of different concentrations (10–40 ppm) and sonicated for 3 min. Prior to irradiation, the suspension was magnetically stirred in the dark for 60 min to establish an adsorption–desorption equilibrium. Then the solution was exposed to visible light by continuous stirring. After the lamp became stable, a fixed amount of H_2_O_2_ solution (0.255 mL, 30 wt%), acting as the electron capture agent, was added into the MB solution. At regular time intervals, an aliquot of 1.5 mL was sampled and centrifuged to measure the concentration of MB by means of the UV/vis spectrometer at the characteristic wavelength of 664 nm.

### 2.4. Detection of hydroxyl radicals

A series of tests were conducted to probe the active species responsible for the photodegradation of MB in the reaction system. ·OH was believed to be one of the important active species during the photodegradation process [29]. As a result, terephthalic acid (TA) was used as a fluorescence probe to detect the ·OH in this study because the TA would easily react with the ·OH and form 2-hydroxyterephthalic acid, a highly fluorescent product. The concentration of TA solution was 0.5 mM in a 2.0 mM NaOH aqueous solution. Under this experimental condition, the hydroxylation reaction process in this system was attributed mainly to ·OH radicals [30]. The setup for the illuminating system and sampling intervals was similar to that for the photodegradation experiments. After sampling, the liquid sample was filtered through a 0.45 *μ*m membrane and analyzed by a fluorescence spectrophotometer. When excited by a wavelength of 315 nm, 2-hydroxyterephthalic acid, the product of TA hydroxylation, exhibited a peak at a wavelength of about 425 nm. Note that considering the rapid generation of ·OH in the system, fluorescence detection of ·OH should be undertaken immediately. At the same time, methanol (*V*(CH_3_OH/H_2_O) = 1:4) was used as a powerful scavenger of ·OH to ascertain the existence of other active species [31]. The other experimental conditions were the same as the photodegradation experiments.

## 3. Results and discussion

### 3.1. Structural properties

**Figure 1.**
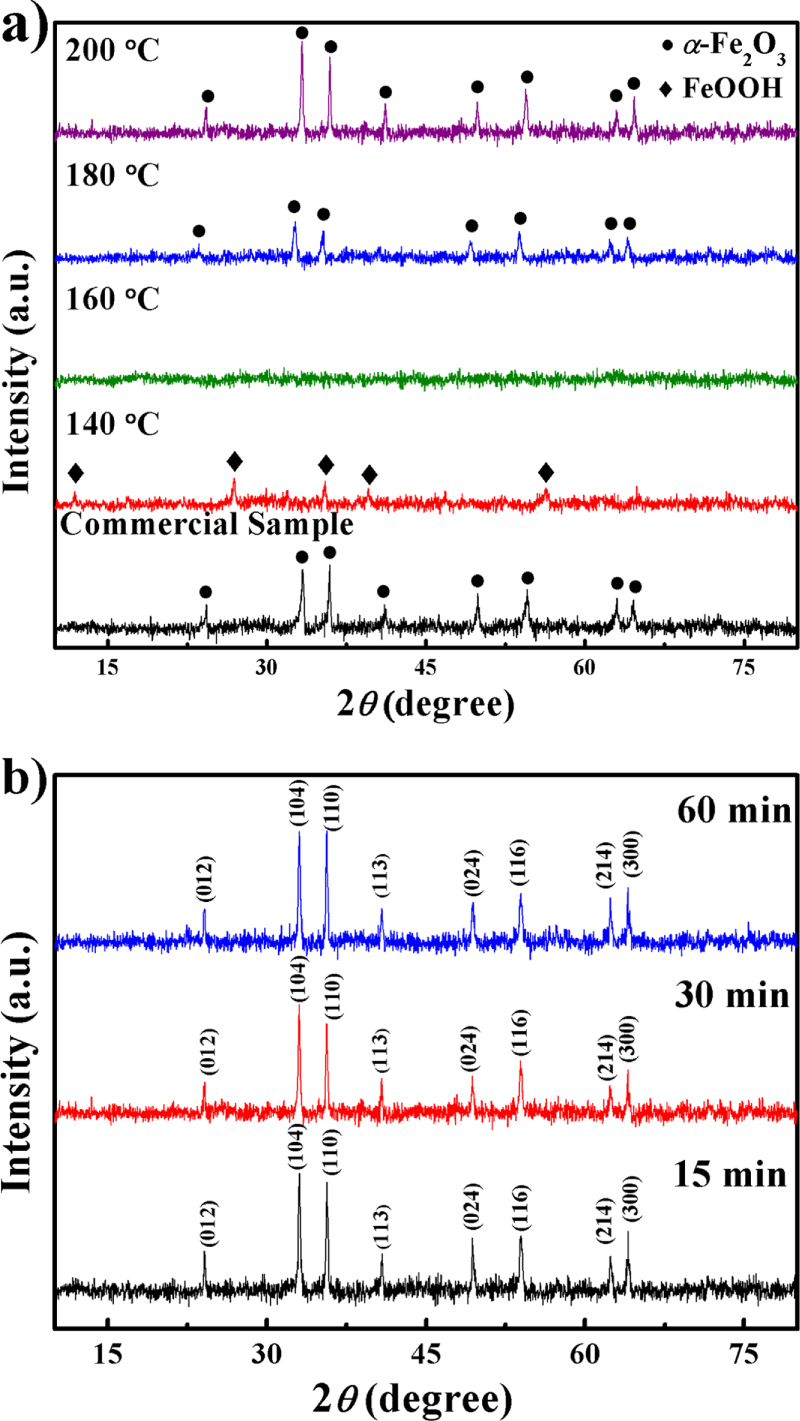
XRD patterns of samples: (a) synthesized at different temperatures; (b) synthesized at 200 °C and different durations.

The XRD patterns of commercial and synthesized Fe_2_O_3_ (140 °C, 160 °C, 180 °C, and 200 °C) are shown in figure 1(a). From the diffraction peaks, it is obvious that as the synthesis temperature increases, the as-synthesized samples change from crystalline to amorphous and then to crystalline again. The observed reflection peaks of the 140 °C sample are in good agreement with the standard pattern of pure FeOOH (JCPDS Card No. 01-0662), whereas the reflection peaks of the 180 °C and 200 °C samples belong to *α*-Fe_2_O_3_ (JCPDS Card No. 24-0072). However, the differences at full-width at half-maximum (FWHM) and relative intensities between the 180 °C and 200 °C samples are apparent. Moreover, compared with the 200 °C sample, the (104) peak is slightly shifted and the (113) peak is missing in the 180 °C sample, suggesting that better crystallinity could be achieved at a higher temperature. According to the well-known Scherrer equation, the average crystallite sizes of 140 °C, 180 °C, and 200 °C were estimated to be 56 nm, 47 nm, and 68 nm, respectively (calculated with the main reflection peak at 2*θ* = 26.734° for the 140 °C sample, 33.0377° for the 180 °C sample, and 33.0522° for the 200 °C sample; table 1). In order to make a comparison, the particle size of commercial Fe_2_O_3_ was also calculated.

**Table 1.** Physical and structural properties of the samples.

Sample	*D*_cryst_^a^ (nm)	SSA^b^ (m^2^g^−1^)	*D*_p_^c^ (nm)	Reaction rate constants *k*^d^ (min^−1^)	Normalized rate constant *k*_*s*_^e^ (×10^−3^ min^−1^ Lm^−2^)	*E*_*g*_ (eV)
140/30	56	—	—	0.035	—	2.00
160/30	—	174.6	2.97	0.033	0.94	1.91
180/30	47	61.02	2.97	0.063	5.16	1.93
200/15	68	28.85	3.08	0.087	15.08	1.94
200/30	68	35.61	2.88	0.083	11.65	1.95
200/60	47	35.62	2.88	0.072	10.10	1.95
200/15/400	79	—	—	—	—	2.05
200/15/450	79	—	—	—	—	2.02
200/15/500	68	—	—	—	—	2.01
Commercial	47	15.19	5.05	0.029	9.54	2.05

a *D*_cryst_ is the crystal domain size determined from x-ray line broadening by using the Scherrer equation.

b SSA is the specific surface area evaluated by using the BET method.

c *D*_*p*_ is the BJH mean pore size obtained on the desorption branch.

d *k* is the apparent reaction rate constant calculated from a pseudo-first-order kinetic model.

e *k*_*s*_ is the rate constant *k* normalized to SSA, *k*_*s*_ = *k* (catalyst concentration × SSA)^−1^.

Application of microwaves significantly accelerated the rates of reaction [32] and crystallization [33] as compared with traditional hydrothermal methods. Figure 1(b) shows XRD patterns of *α*-Fe_2_O_3_ nanoparticles microwave-treated at 200 °C for 15, 30, and 60 min. The synthesis time did not significantly affect crystallinity and particle size (table 1). Since no obvious difference could be determined by the XRD analysis after 15 min synthesis, it is suggested that the reaction and crystallization process can be accomplished in an extremely short time, whereas the traditional hydrothermal method requires dozens of hours. Without question, efficiency can be greatly improved.

Annealing treatment is generally believed to improve crystallinity and is therefore usually carried out after the hydrothermal process. As shown in this study, with an additional annealing treatment, the crystallinity of *α*-Fe_2_O_3_ is greatly improved (figure S1(a), supporting information), with a size distribution around 68 to 79 nm, indicating more uniform crystallization and lower defect concentration than in as-grown samples.

**Figure 2.**
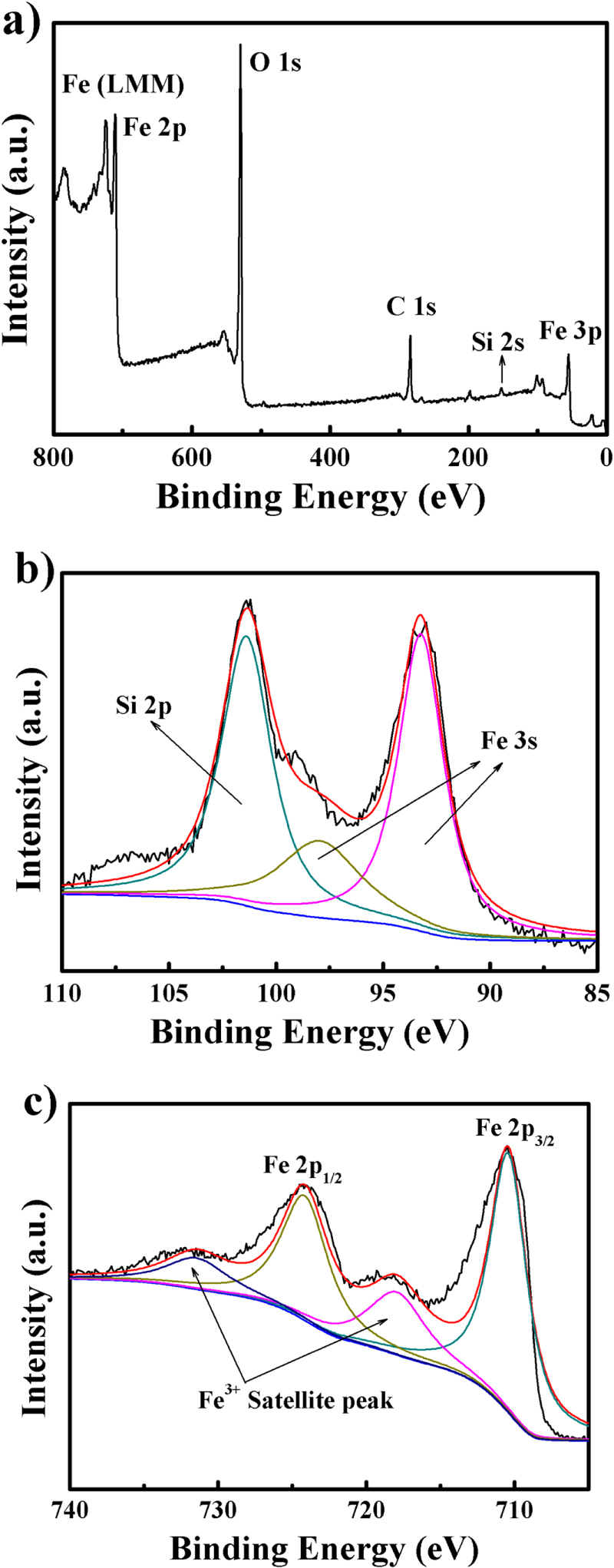
(a) XPS spectra of a *α*-Fe_2_O_3_ nanodisk; (b) high resolution of the Si 2p peak; (c) high resolution of the Fe 2p peak.

Even though silicate anions are used in the precursor solution, no diffraction peaks of Si oxide could be observed in any of the XRD patterns due to the small residue of silicate anions in the *α*-Fe_2_O_3_. As examined by XPS, evidence of the existence of Si was obtained, as shown in figure 2(b). Si 2s and Si 2p peaks could be observed in the sample, with a lower binding energy compared with SiO_2_, indicating the oxygen bridge between Si and Fe atoms, which is consistent with previous reports [28, 34]. The Fe 2p spectrum (figure 2(c)) exhibits the typical 2p_1/2_ and 2p_3/2_ peaks of Fe^3+^ at 724.4 eV and 710.8 eV, with characteristic satellite peaks at 718.8 eV and 732.5 eV [24, 35, 36]. Moreover, the O 1s core levels show dominant oxide peaks at around 531.11 eV, inconsistent with the O^2−^ state [36]. Therefore, it is confirmed that the sample is pure *α*-Fe_2_O_3_ with some Si atoms acting as the oxygen bridges. It is reported that Si [37], Ti [38], Mo [39], Cr [39], and Sn [40] can improve electron-hole separation. With the residual Si in *α*-Fe_2_O_3_, it is reasonable to expect a better photocatalytic performance in dye degradation. Generally, there should be an optimum amount of Si to achieve the best performance. In accordance with this, research into delicate control of the Si in nanodisks is under way.

### 3.2. Morphology

**Figure 3.**
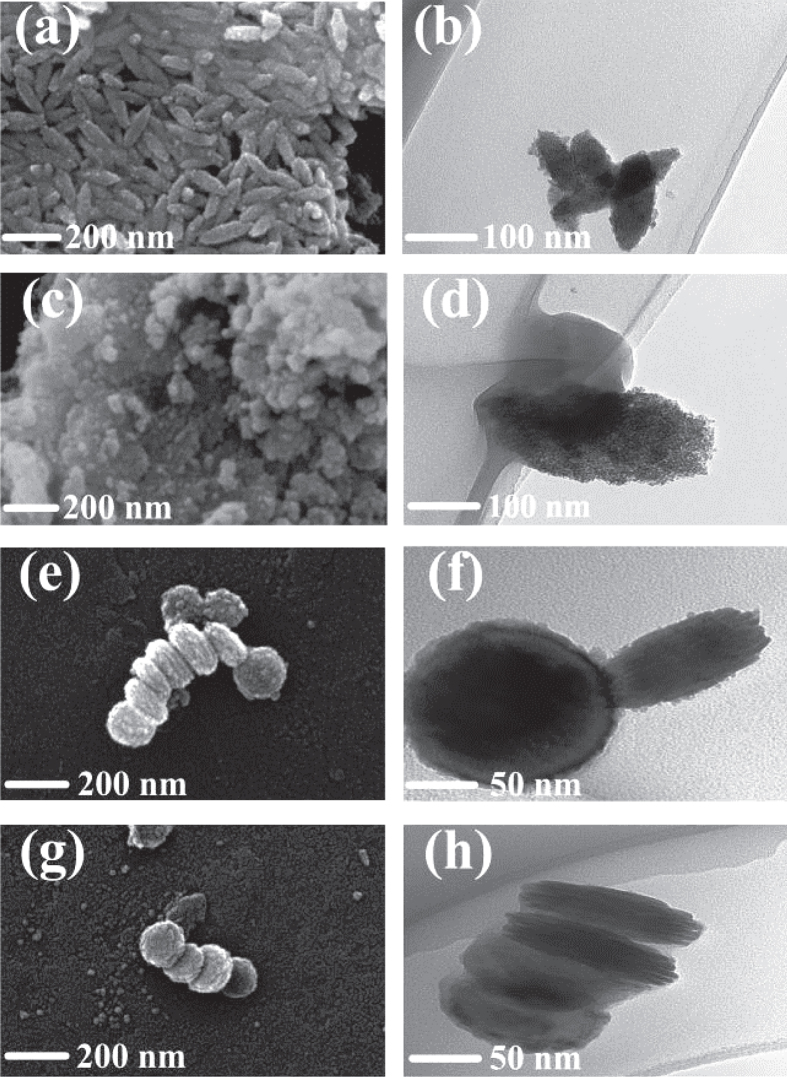
SEM and TEM analysis of samples synthesized at different temperatures: (a) and (b) 140 °C, nanospindle; (c) and (d) 160 °C, amorphous nanoparticle; (e) and (f) 180 °C, pseudo-nanodisk; (g) and (h) 200 °C, uniform nanodisk.

The morphology of *α*-Fe_2_O_3_ synthesized at different temperatures in an aqueous medium of anhydrous iron chloride and sodium silicate additive shows a series of distinct structures: nanospindle, amorphous nanoparticle, pseudo-multilayer nanodisk, and highly symmetrical multilayered nanodisk, as shown in figures 3(a)–(h). Interestingly, the various structures are in line with the corresponding XRD patterns, demonstrating a transformation that evolves with increasing synthesis temperature: from crystalline to amorphous and then to crystalline again. Dramatically, the corresponding TEM image of amorphous nanoparticles (figure 3(d)) shows a pseudo-spindle shape with a blunt tip, indicating that at this temperature range Ostwald ripening plays the key role in nucleation instead of oriented attachment [41, 42], which might be the main reason for the amorphous structure.

Once the synthesis temperature is higher than 180 °C, *α*-Fe_2_O_3_ samples transform into multilayer nanodisk shapes (figures 3(e)–(h)). Meanwhile, the nanodisk becomes more uniform. This is mainly owing to the advantages of the microwave-assisted method: homogenous heating without a temperature gradient and rapid nucleation with a uniform structure [33]. Figure 3(h) shows a typical TEM image of the multilayer structure in a nanodisk. It can be clearly seen that each nanodisk, with a relatively rough surface, has a diameter around 100 nm and a thickness around 60 nm. Moreover, a layer structure (6 to 10 layers) can be observed in the nanodisk. The proposed mechanism of the nanodisk formation process is illustrated in scheme 1 of the supporting information. First, with the aid of microwaves, the crystallization of nanoparticles completes rapidly. Subsequently, with continuous microwave-assisted heating, the nanoparticles self-assemble into multilayered nanodisks.

**Figure 4.**
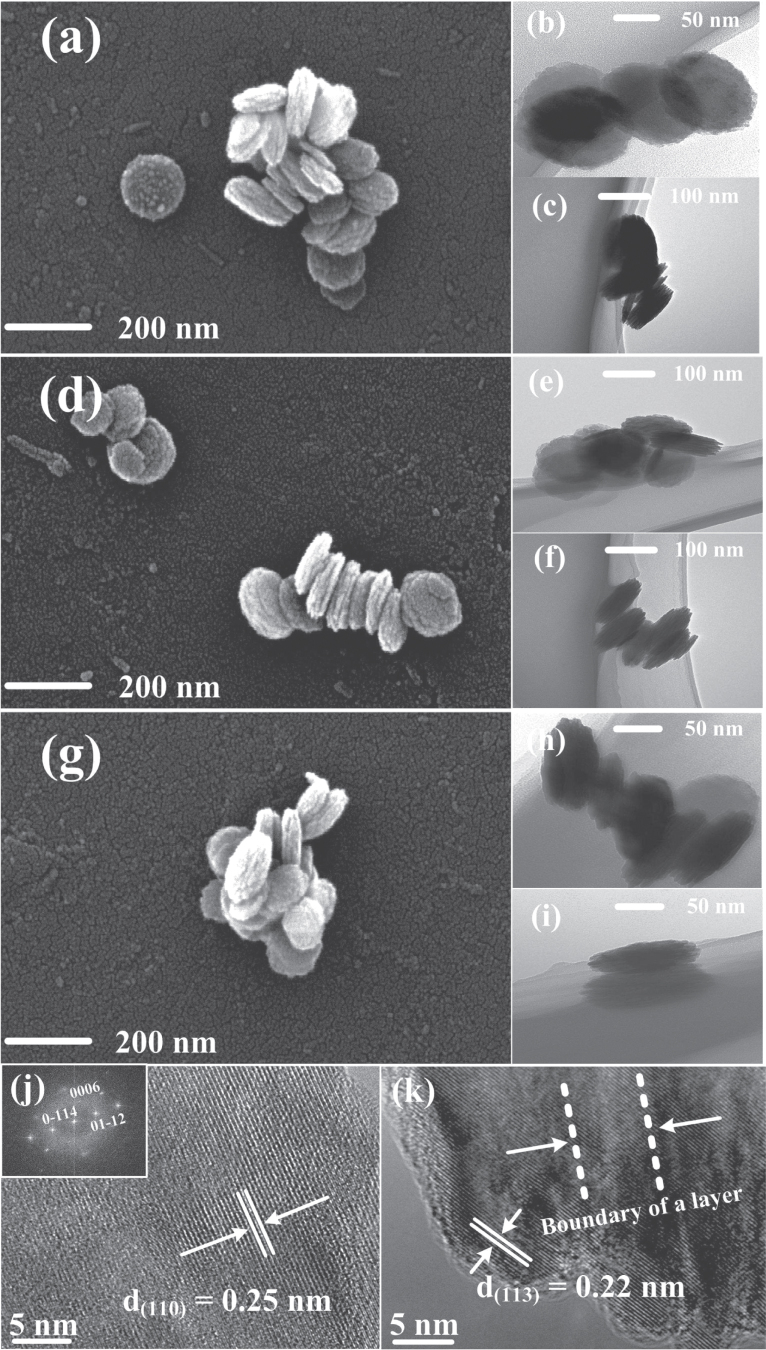
SEM and TEM analysis of *α*-Fe_2_O_3_ nanodisks synthesized at 200 °C within: (a), (b), and (c) 15 min; (d), (e), and (f) 30 min; (g), (h), and (i) 60 min. (j) and (k) Top-view and side-view HRTEM images, respectively (inset: FTT pattern).

The morphology of *α*-Fe_2_O_3_ nanodisks synthesized with microwave irradiation for 15, 30, and 60 min is shown in figures 4(a)–(k). All nanodisks exhibit round shapes with a diameter of approximately 100 nm (figures 4(a), (d), and (g)) and multilayer structures with a thickness of approximately 60 nm (figures 4(c), (f), and (i)). These results agree with the particle size calculated from the XRD patterns. Figure 4(j) is the HRTEM image acquired from the top view of a single *α*-Fe_2_O_3_ nanodisk. The lattice fringes are aligned perfectly across the surface with a spacing of 0.25 nm corresponding to the (110) planes, confirming the highly crystalline *α*-Fe_2_O_3_. It is worth noting that each adjacent layer of the nanodisks is around 6 nm, as shown in the HRTEM side-view image (figure 4(k)). It is expected that this special multilayered structure would be favorable for the separation of excited electron-hole pairs since the thickness is close to the intrinsic diffusion length (2–4 nm). Therefore, the photodegradation performance is enhanced.

The morphology of a *α*-Fe_2_O_3_ nanodisk with an additional annealing treatment (400 °C, 450 °C, and 500 °C) is shown in figures S1(b)–(d) (Supporting Information). Even though the nanodisks have good uniformity, they tend to aggregate as a globular shape, which is probably the reason for the poor photodegradation performance (figure S2, supporting information).

### 3.3. Photocatalytic activity

**Figure 5.**
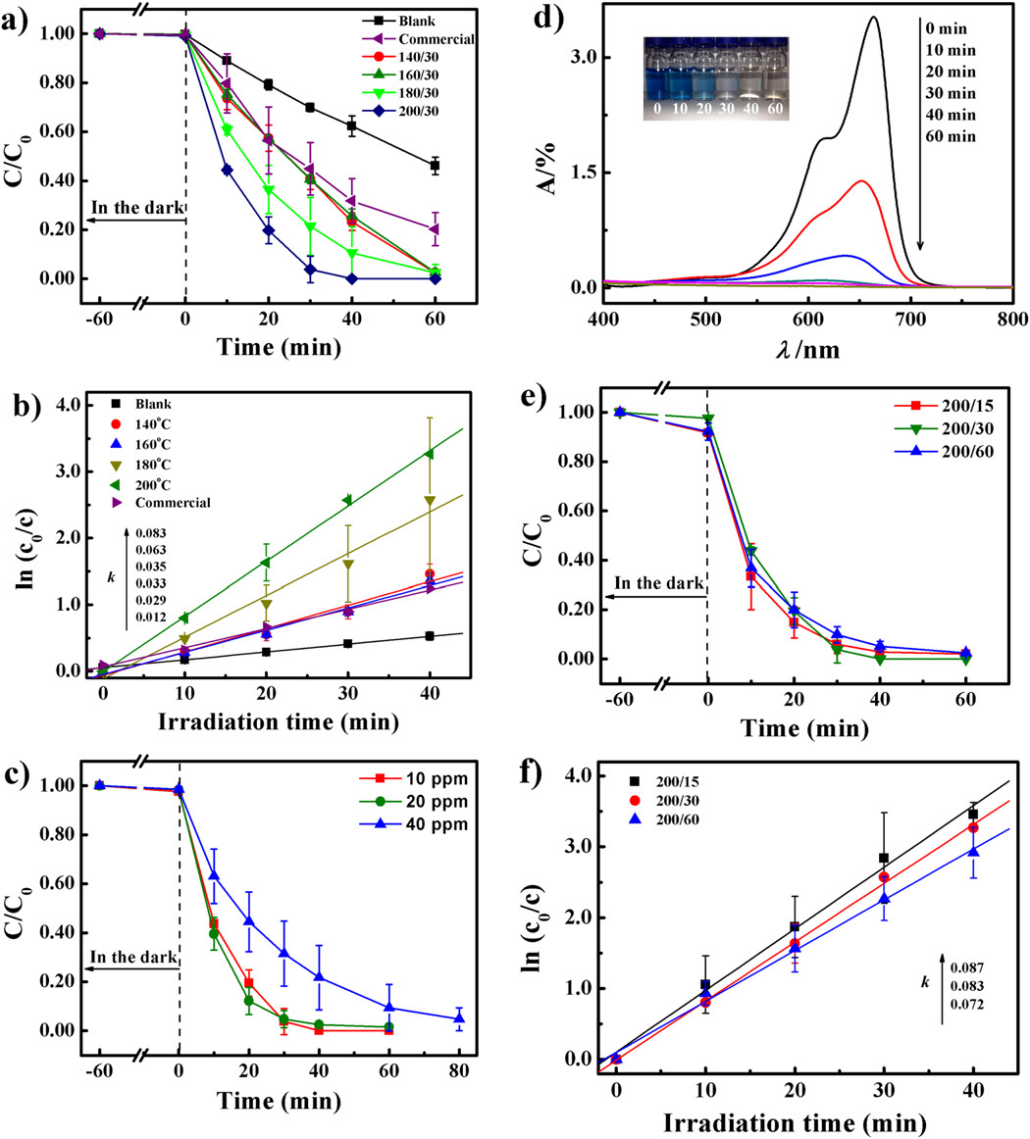
(a) MB degradation as a function of irradiation time with different as-synthesized samples; (b) the kinetic analysis (pseudo-first-order) of different as-synthesized samples; (c) the effect of MB concentration on MB degradation with a 200/30 sample; (d) UV/vis spectral changes of 20 ppm MB aqueous solution in the presence of a 200/30 sample and images (inset) of the aqueous solution recorded at different time intervals; (e) MB degradation as a function of irradiation time; (f) kinetic analysis (pseudo-first-order) for different durations of microwave irradiation during sample synthesis.

As revealed in the SEM and TEM images, the morphology changes with increasing synthesis temperature. Since absorption and electron transfer depend greatly on the surface structure of a nanocrystal, it is expected that the nanocrystals prepared at different temperatures should present different photocatalytic activities. Figure 5(a) shows the photocatalytic activities of samples with different synthesis temperatures. The degradation efficiency is defined as *c*/*c*_0_, where *c*_0_ and *c* are the initial (after equilibrium adsorption) and reaction concentrations, respectively, of MB at a specific time. It can be seen that, compared with the blank experiment (without addition of *α*-Fe_2_O_3_ photocatalyst but only H_2_O_2_), the as-synthesized and commercial samples show much better photocatalytic activities. Moreover, it is found that among all the samples, the 200 °C synthesized *α*-Fe_2_O_3_ nanodisk shows superior photocatalytic activity: more than 90% of MB can be degraded in 30 min, whereas the degradation efficiency of the commercial sample is only 50%. The reaction rate constant *k* of the as-synthesized and commercial samples is shown in figure 5(b) and table 1. It is evident that the 200 °C synthesized *α*-Fe_2_O_3_ nanodisk has the highest reaction rate constant, 0.087 min^−1^. When normalized to the Brunauer–Emmett–Teller surface areas (S_BET_), it is 15.08 × 10^−3^ min^−1^ Lm^−2^, which is also highest among all the samples (table 1). It is evident that the photocatalytic performance is comparable with previous reported *α*-Fe_2_O_3_ nanoplate structures (*k* = 0.008) or SnO_2_/*α*-Fe_2_O_3_ hierarchical nano-heterostructures (*k* = 0.011) [23, 28].

Figure 5(c) shows the effect of different initial concentrations on the degradation of MB, and the UV/vis spectra of the samples collected at various time intervals for the concentration of 20 ppm are shown in figure 5(d). It is clearly shown that after 20 min of irradiation the main absorbance of MB at 664 nm decreases dramatically, indicating that the degradation efficiency does not obviously decrease. However, once the concentration increases to 40 ppm, it is apparent that the degradation efficiency decreases; this can be elucidated as follows: (1) if dye concentration increases continuously, the dye molecules will increase, which will shield part of the visible light and make it difficult to trigger the photocatalyst; (2) the reaction sites of the photocatalyst will also be covered by the excess dye molecules, leading to a reduction in the produced hydroxyl radicals [23, 31].

As previously mentioned, a longer microwave irradiation time will not obviously improve morphology and crystallinity. Therefore, it is reasonable to anticipate similar photocatalytic activities in the samples synthesized for different durations. Indeed, figures 5(e) and (f) show similar photocatalytic efficiencies and reaction constants. However, the photocatalytic activities of the annealed samples decrease significantly (figure S2, supporting information), even though they possess better crystallinity and uniform morphology. As mentioned, aggregation occurred during the annealing treatment, resulting in a decrease in active reaction sites, which is the main reason for this phenomenon.

### 3.4. Discussion of mechanisms

#### 3.4.1. Enhanced visible-light light absorption

**Figure 6.**
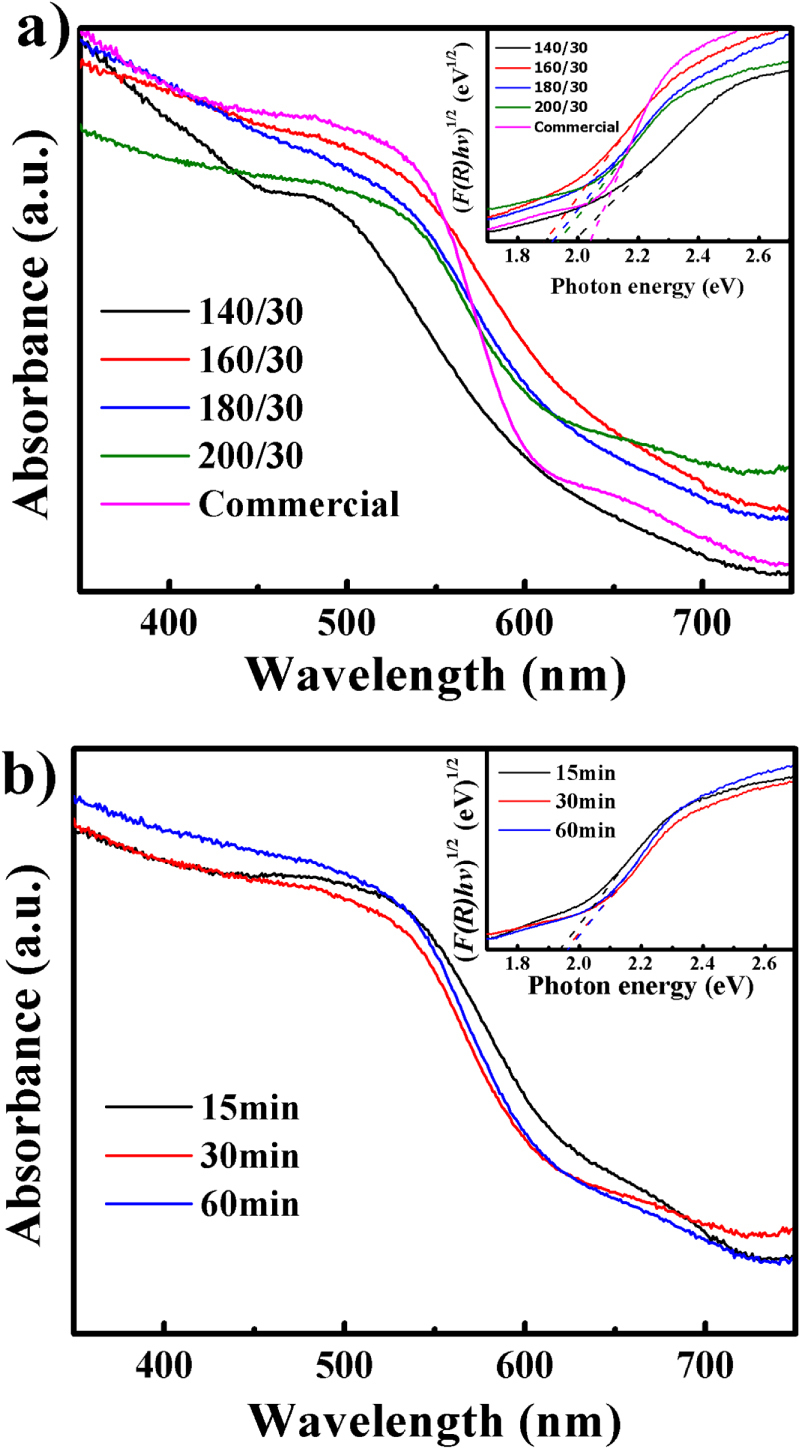
UV/vis DRS spectra of samples synthesized (a) at different temperatures and (b) for different durations at 200 °C. The insets of (a) and (b) show the plots of the (*F*(*R*)*hv*)^2^ versus photon energy (*hv*) for the photocatalyst.

The high photocatalytic activity is first attributed to the large absorption range of light, which is crucial for good photocatalytic activity, especially for visible-light degradation. Figure 6 shows the UV/Vis DRS of the microwave-synthesized samples and the commercial sample. As shown in figure 6(a), there is a clear red shift of about 10–30 nm in the absorption edge of the microwave-synthesized samples (180 °C and 200 °C) compared with the commercial samples, which is attributed to the multilayer plate structure [43]. The curves of (*F*(*R*)*hν*)^2^ versus (*hv*) for the samples are also plotted in the inset of figure 6(a). Through extrapolation of the linear portions of these curves to *F*(*R*) = 0, the *E*_*g*_ values of the commercial and microwave samples are obtained and shown in table 1. It is obvious that the *E*_*g*_ of the microwave-synthesized samples is smaller than that of the commercial sample. As a result of a smaller *E*_*g*_, an extended photoresponse range of approximately 640–670 nm could be achieved, leading to more efficient utilization of the solar spectrum. Thus, the microwave-synthesized samples show significant improvement in MB degradation in the visible-light region. For samples synthesized within 15, 30, and 60 min, the UV/Vis DRS and *E*_*g*_ are similar (figure 6(b) and table 1), indicating that the photocatalytic activities of the samples should be similar. This inference can be confirmed by the MB degradation results (figures 5(e) and (f)). Furthermore, the similarity indicates that these three samples should have almost the same size and structure, which is in good agreement with the SEM and TEM results shown in figures 4(a)–(j).

As mentioned earlier, after annealing treatment, the *α*-Fe_2_O_3_ nanodisks tend to aggregate spontaneously, which should be one of the reasons for the poor photocatalytic performance. In addition, as shown in the inset of figure S3 (supporting information), the *E*_*g*_ of the annealed samples increases up to 2.05 eV, resulting in lower absorption of visible light, which should be another reason for lower photocatalytic activity.

#### 3.4.2. Efficient electron-hole separation

**Figure 7.**
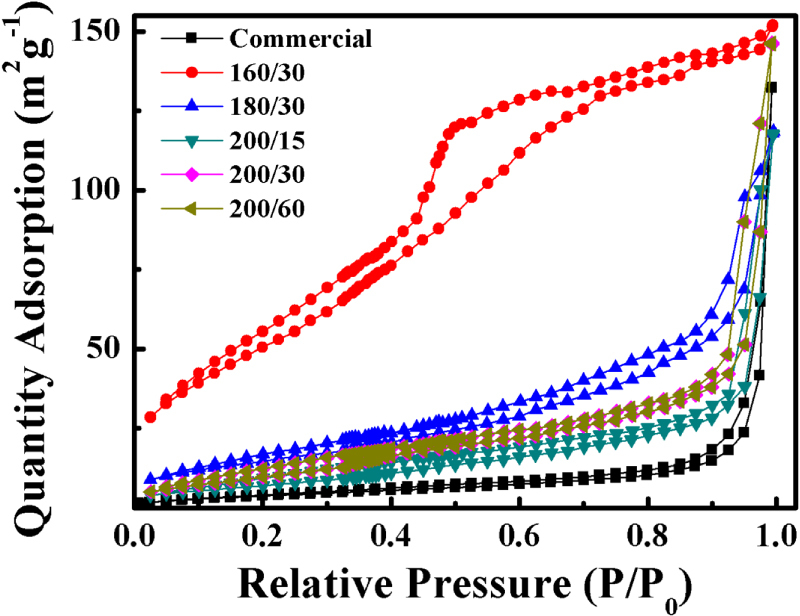
Nitrogen adsorption/desorption isotherms.

The N_2_ adsorption/desorption isotherm of the samples exhibits a representative type IV isotherm with pore sizes around 3 nm, as shown in figure 7 and table 1. It is reasonable to attribute the high photocatalytic activity to enhanced adsorption brought about by the high S_BET_. However, through the results of this study, adsorption can be excluded as the main mechanism during MB degradation due to the unobvious change in MB concentration after the 60 min dark adsorption process. The S_BET_ of the nanodisk is calculated to be 28.85 m^2^g^−1^, which is the smallest among the as-synthesized samples (table 1). Despite this, the nanodisk shows the highest photocatalytic activity (figure 5(a)), demonstrating that key factors other than adsorption are contributing to the enhanced photocatalytic activity.

**Figure 8.**
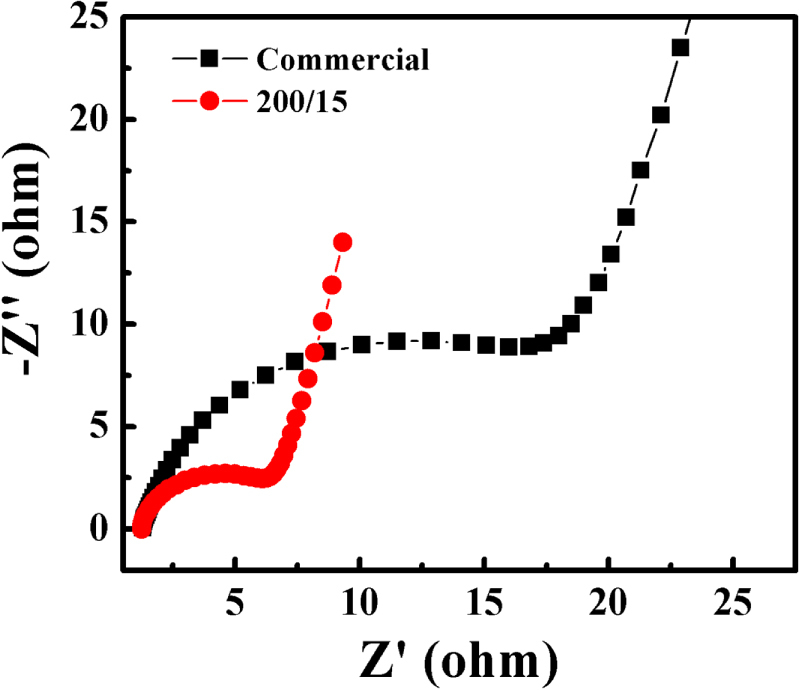
EIS changes in *α*-Fe_2_O_3_ nanodisk and commercial sample electrodes.

Because of the intrinsic short hole diffusion length (2–4 nm) [17, 18], photogenerated electron-hole pairs in *α*-Fe_2_O_3_ cannot be easily separated, resulting in an unsatisfied photocatalytic performance in some samples (140 °C, 160 °C, 180 °C, and commercial). However, the *α*-Fe_2_O_3_ nanodisk is combined with a few nanoplates with a thickness around 3–6 nm (figure 4). As a result of the thin-layered structure, electron-hole pairs can separate and participate in the photo-oxidation process on the surface more effectively. To further confirm the effective separation of electron-hole pairs in the *α*-Fe_2_O_3_ nanodisk, EIS was conducted, as shown in figure 8. Only one semicircle was observed on the EIS plane, suggesting that the photocatalytic reaction involved only the surface charge-transfer process. The semicircle radius of the *α*-Fe_2_O_3_ nanodisk electrode is much smaller than that of the commercial sample, indicating a decrease in the solid-state interface layer resistance. This leads to a higher transfer rate of the electron-hole pairs [44]. As a result, aggregation of the electrons is alleviated, thus reducing their recombination rate. The separation and transmission of electron-hole pairs should be improved, which is crucial to photocatalytic performance [45]. Furthermore, the residual Si as dopant may also improve the photoelectrolysis and electrical properties [37], another potential factor in high photocatalytic activity. Therefore, an *α*-Fe_2_O_3_ nanodisk with a thin-layer structure presents high photocatalytic activity.

#### 3.4.3. Effect of scavenger agents and investigation on active species

In the presence of H_2_O_2_, the electrons generated by *α*-Fe_2_O_3_ can be trapped easily and form ·OH radicals, which can enhance photocatalytic activity remarkably [46–48]. This can be understood by the following equations:1
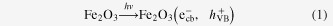

2
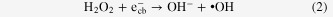

3
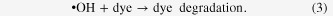



In addition, the generated electrons can also be trapped by the surface of Fe^3+^ and form Fenton’s reagent [49, 50]. The reactions are described in the following equations:4
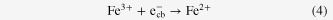

5
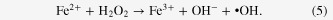



The ·OH radicals formed in equations (2) and (5) are the triggers of photocatalytic reaction, which promotes the degradation of MB, as shown in equation (3).

**Figure 9.**
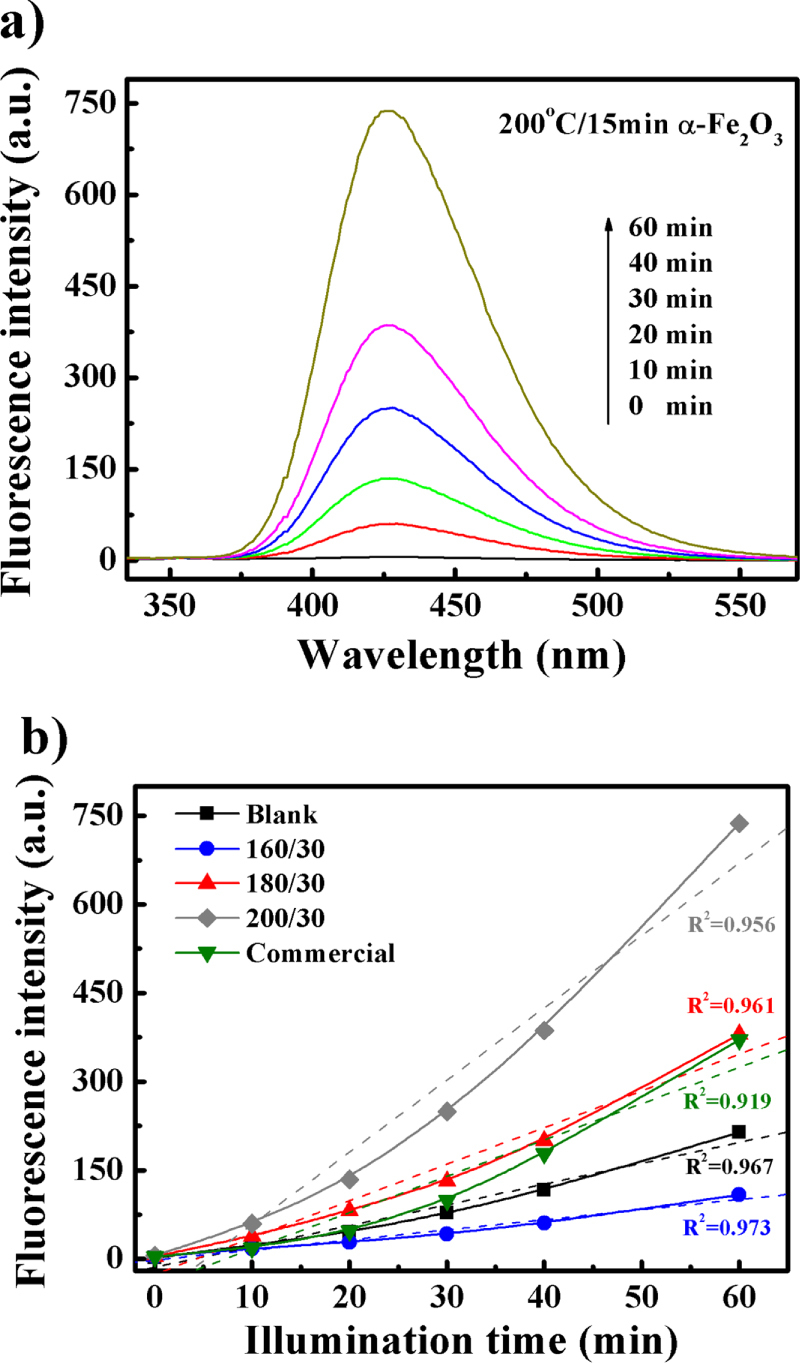
Fluorescence quenching to detect OH: (a) full wavelength scan of 200/15 sample and (b) different as-synthesized samples.

Due to its unique multilayered structure, electron-hole pairs can separate in an *α*-Fe_2_O_3_ nanodisk more effectively. As a result, enhanced production of ·OH radicals can be expected. As shown in figure 9(a), after 10 min irradiation, the fluorescence intensity of the 200/15 sample obviously increases, indicating increased production of ·OH radicals in the system. In addition, the fluorescence intensity of the ·OH radicals increases with irradiation time, which is consistent with the photocatalytic activity shown in figure 5(d). The fluorescence spectra of other as-synthesized samples are shown in figure 9(b). The order of the ·OH radicals produced is found to be 200/30 > 180/30 ≈ commercial > blank > 160/30, which seems contradictory to the photodegradation results (figure 5(a)). However, recent studies have pointed out that the role of ·OH radicals in photodegradation is probably overestimated [29, 51–54] in terms of other active oxygen species might also be generated during the irradiation process and contribute to photocatalytic degradation. The generation of other active oxygen species might be the reason for this strange phenomenon. In addition, the number of ·OH radicals produced performed a zero-order kinetic model according to a previous study [55]. However, as shown in figure 9(b), the production of ·OH radicals fits with rather a second-order kinetic model as opposed to zero order in this study. Therefore, it is reasonable to assert that other oxygen species might also contribute to dye degradation.

**Figure 10.**
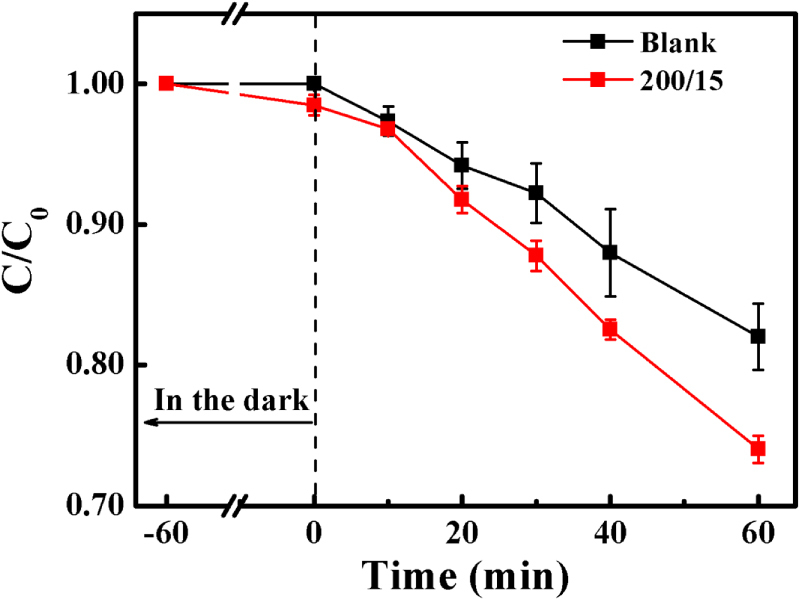
The effect of methanol as a scavenger agent on MB degradation.

Figure 10 shows that when methanol is put into the degradation system, a significant decrease in MB degradation occurs in both the blank and the 200/15 sample, indicating that ·OH radicals should be the main oxidizing agent. However, the 200/15 sample still shows better photocatalytic degradation (∼25%) than the blank sample with the existence of a scavenger. Thus other active oxygen species must be produced during the photocatalytic reaction. O^·2−^ is usually reported to be one of the probable oxidizing agents, and its formation and photocatalytic reaction are demonstrated in equations (6) and (7):6
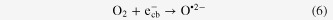

7
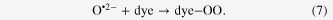



From these equations, it is clear that the adsorbed O_2_ will capture the photogenerated electrons and form O^·2−^, which can degrade dye directly. Moreover, O^·2−^ can also react with the photogenerated holes and then form active ·OH radicals and peroxides. Or it can react with the scavengers and form organic peroxides. The reactions are demonstrated in equations (8) and (9):8
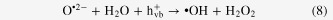

9
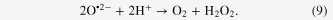



Based on the foregoing reaction mechanism, the importance of O^·2−^ to dye degradation is conspicuous. Research into the relationship between adsorbed O_2_ in a dye solution and O^·2−^ production is under way. In summary, it can be inferred that the main active species is ·OH, whereas O^·2−^ only partly contributes to MB degradation.

## 4. Conclusions

In summary, remarkably enhanced electron-hole separation and visible-light photocatalytic performance were observed in *α*-Fe_2_O_3_ nanomaterial with disk morphology. Microwave-assisted fabrication with a significantly reduced synthesis period was found to play an important role in the formation of multilayer nanodisk structures, which effectively suppress the well-known problem of short hole diffusion length in *α*-Fe_2_O_3_ materials. In addition, a comprehensive mechanism analysis indicates that ·OH radicals dominate in the photodegradation system, and the generation of other active oxygen species was also demonstrated. Based on the results, it is reasonable to expect that microwave-synthesized *α*-Fe_2_O_3_ nanodisks can be a promising photocatalyst for various environmental applications.

## References

[C1] Albu S P, Ghicov A, Macak J M, Hahn R, Schmuki P (2007). Nano Lett..

[C2] Zheng Y, Chen C, Zhan Y, Lin X, Zheng Q, Wei K, Zhu J, Zhu Y (2007). Inorg. Chem..

[C3] McLaren A, Valdes-Solis T, Li G, Tsang S C (2009). J. Am. Chem. Soc..

[C4] Vernardou D, Drosos H, Spanakis E, Koudoumas E, Savvakis C, Katsarakis N (2011). J. Mater. Chem..

[C5] Liu Y, Yu L, Hu Y, Guo C, Zhang F, Wen Lou X (2012). Nanoscale.

[C6] Niu M, Huang F, Cui L, Huang P, Yu Y, Wang Y (2010). ACS Nano.

[C7] Yu H, Chen S, Quan X, Zhao H, Zhang Y (2008). Environ. Sci. Technol..

[C8] Tao J, Luttrell T, Batzill M (2011). Nat. Chem.

[C9] Chen X, Liu L, Yu P Y, Mao S S (2011). Science.

[C10] Xia Y, Yin L (2013). Phys. Chem. Chem. Phys..

[C11] Zuo F, Wang L, Wu T, Zhang Z, Borchardt D, Feng P (2010). J. Am. Chem. Soc..

[C12] Hoang S, Berglund S P, Hahn N T, Bard A J, Mullins C B (2012). J. Am. Chem. Soc..

[C13] Wang C, Meng D, Sun J, Memon J, Huang Y, Geng J (2014). Adv. Mater. Interfaces.

[C14] Tang J, Zou Z, Ye J (2004). Angew. Chem. Int. Edn..

[C15] Yi Z (2010). Nat. Mater..

[C16] Faust B C, Hoffmann M R, Bahnemann D W (1989). J. Phys. Chem..

[C17] Linsebigler A L, Lu G, Yates J T (1995). Chem. Rev..

[C18] Dare-Edwards M P, Goodenough J B, Hamnett A, Trevellick P R (1983). J. Chem. Soc., Faraday Trans..

[C19] Vayssieres L, Sathe C, Butorin S M, Shuh D K, Nordgren J, Guo J (2005). Adv. Mater..

[C20] Mohapatra S K, John S E, Banerjee S, Misra M (2009). Chem. Mater..

[C21] Liu J, Liang C, Zhang H, Zhang S, Tian Z (2011). Chem. Commun..

[C22] Chen L, Yang X, Chen J, Liu J, Wu H, Zhan H, Liang C, Wu M (2010). Inorg. Chem..

[C23] Zhang S, Li J, Niu H, Xu W, Xu J, Hu W, Wang X (2013). ChemPlusChem.

[C24] Hu X, Yu J C, Gong J, Li Q, Li G (2007). Adv. Mater..

[C25] Nyutu E K, Chen C-H, Sithambaram S, Crisostomo V M B, Suib S L (2008). J. Phys. Chem. C.

[C26] Qiu G, Dharmarathna S, Genuino H, Zhang Y, Huang H, Suib S L (2011). ACS Catal..

[C27] Opembe N N, King’ondu C K, Espinal A E, Chen C-H, Nyutu E K, Crisostomo V M, Suib S L (2010). J. Phys. Chem. C.

[C28] Qu J, Yu Y, Cao C-Y, Song W-G (2013). Chem. Eur. J..

[C29] Zhao W, Ma W, Chen C, Zhao J, Shuai Z (2004). J. Am. Chem. Soc..

[C30] Ishibashi K-I, Fujishima A, Watanabe T, Hashimoto K (2000). Electrochem. Commun..

[C31] Yin M, Li Z, Kou J, Zou Z (2009). Environ. Sci. Technol..

[C32] Mallikarjuna N N, Varma R S (2007). Cryst. Growth Des..

[C33] Baghbanzadeh M, Carbone L, Cozzoli P D, Kappe C O (2011). Angew. Chem. Int. Edn.

[C34] Guillet-Nicolas R, Bridot J-L, Seo Y, Fortin M-A, Kleitz F (2011). Adv. Funct. Mater..

[C35] Walling C, Johnson R A (1975). J. Am. Chem. Soc..

[C36] Xu J-S, Zhu Y-J (2012). Cryst. Eng. Comm..

[C37] Cesar I, Kay A, Gonzalez Martinez J A, Grätzel M (2006). J. Am. Chem. Soc..

[C38] Wang G, Ling Y, Wheeler D A, George K E N, Horsley K, Heske C, Zhang J Z, Li Y (2011). Nano Lett..

[C39] Kleiman-Shwarsctein A, Hu Y-S, Forman A J, Stucky G D, McFarland E W (2008). J. Phys. Chem. C.

[C40] Ling Y, Wang G, Wheeler D A, Zhang J Z, Li Y (2011). Nano Lett..

[C41] Liu B, Zeng H C (2005). Small.

[C42] Challa S R, Delariva A T, Hansen T W, Helveg S, Sehested J, Hansen P L, Garzon F, Datye A K (2011). J. Am. Chem. Soc..

[C43] Wang J, White W B, Adair J H (2005). J. Am. Ceram. Soc..

[C44] He B-L, Dong B, Li H-L (2007). Electrochem. Commun..

[C45] Wang L, Wei H, Fan Y, Gu X, Zhan J (2009). J. Phys. Chem. C.

[C46] Yu J, Yu X, Huang B, Zhang X, Dai Y (2009). Cryst. Growth Des..

[C47] Stroyuk A L, Shvalagin V V, Kuchmii S Y (2005). J. Photochem. Photobiol. A.

[C48] Serpone N, Lawless D, Khairutdinov R (1995). J. Phys. Chem..

[C49] Esplugas S, Giménez J, Contreras S, Pascual E, Rodrı´guez M (2002). Water Res..

[C50] Zhang Y-G, Ma L-L, Li J-L, Yu Y (2007). Environ. Sci. Technol..

[C51] Yang J, Chen C, Ji H, Ma W, Zhao J (2005). J. Phys. Chem. B.

[C52] Carter E, Carley A F, Murphy D M (2007). J. Phys. Chem. C.

[C53] Komaguchi K, Maruoka T, Nakano H, Imae I, Ooyama Y, Harima Y (2009). J. Phys. Chem. C.

[C54] Wang Z, Ma W, Chen C, Ji H, Zhao J (2011). Chem. Eng. J..

[C55] Xiao Q, Si Z, Zhang J, Xiao C, Tan X (2008). J. Hazard. Mater..

